# Genetic Pathways of Neuroregeneration in a Novel Mild Traumatic Brain Injury Model in Adult Zebrafish

**DOI:** 10.1523/ENEURO.0208-17.2017

**Published:** 2018-01-03

**Authors:** Amanda L. Maheras, Brian Dix, Olivia M. S. Carmo, Aleena E. Young, Vanessa N. Gill, Julia L. Sun, Aleah R. Booker, Helen A. Thomason, Anastasia E. Ibrahim, Lauren Stanislaw, Jennifer C. Dallego, Cat N. Ngo, Audrey Chen, Barbara K. Fortini, Rory D. Spence

**Affiliations:** 1 Scripps College, Claremont, CA 91711; 2 Claremont McKenna College, Claremont, CA 91711; 3 Keck Science Department, Claremont, CA 91711; 4 Harvey Mudd College, Claremont, CA 91711; 5 Ayala School of Biological Sciences, Department of Neurobiology & Behavior, University of California, Irvine, Irvine, CA; 6 School of Pharmacy, Keck Graduate Institute, Claremont, CA 91711

**Keywords:** Concussion, DEG, mTBI, RNA-seq, TBI, zebrafish

## Abstract

Mild traumatic brain injuries (mTBIs) are one of the most prevalent neurological disorders, and humans are severely limited in their ability to repair and regenerate central nervous system (CNS) tissue postinjury. However, zebrafish (*Danio rerio*) maintain the remarkable ability to undergo complete and functional neuroregeneration as an adult. We wish to extend knowledge of the known mechanisms of neuroregeneration by analyzing the differentially expressed genes (DEGs) in a novel adult zebrafish model of mTBI. In this study, a rodent weight drop model of mTBI was adapted to the adult zebrafish. A memory test showed significant deficits in spatial memory in the mTBI group. We identified DEGs at 3 and 21 days postinjury (dpi) through RNA-sequencing analysis. The resulting DEGs were categorized according to gene ontology (GO) categories. At 3 dpi, GO categories consisted of peak injury response pathways. Significantly, at 21 dpi, GO categories consisted of neuroregeneration pathways. Ultimately, these results validate a novel zebrafish model of mTBI and elucidate significant DEGs of interest in CNS injury and neuroregeneration.

## Visual Abstract

**Figure:**
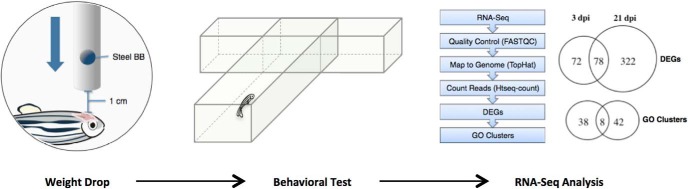

## Significance Statement

Mild traumatic brain injuries (mTBIs) are a major health concern in the United States, with ∼2.8 million concussions reported annually by the CDC. Despite increased awareness within the last decade of the dangers surrounding mTBIs, incidence of concussions continues to rise. Humans are extremely limited in their ability to repair their brain after an injury such as a concussion. To better understand this issue, we developed a novel mTBI model in adult zebrafish. Our model is inexpensive and easily adaptable for other researchers. Much like humans, zebrafish brains undergo an injury response after an injury. Unlike humans, zebrafish maintain the remarkable ability to regenerate and repair their brain after a concussion. We have analyzed the neural pathways involved in zebrafish brain regeneration. These data provide critical insight into the processes of neural repair in zebrafish and will contribute to a better understanding in the scientific community of neuroregeneration.

## Introduction

Traumatic brain injuries (TBIs) are a leading health concern in the United States, contributing to 30% of all reported injury-related deaths. In 2010, there were ∼2.5 million reported cases of TBI in the United States, either from an isolated injury or concurrent with other trauma (Taylor et al., 2017). These numbers are assumed to be grossly underestimated, as many injuries are likely not being reported and/or individuals are not seeking care (Corrigan et al., 2010). An increasing amount of investigation has focused on the occurrence, pathophysiology of initial and secondary injuries, and recovery process of TBIs (Boyle et al., 2014; Taylor et al., 2017). As a result, TBIs are now being treated more as a disease than as a traumatic event (Masel and DeWitt, 2010), including mild TBI (mTBI), more commonly known as a concussion. Initial symptoms of a mTBI may include loss of consciousness, amnesia, headaches, and nausea (Len and Neary, 2011), but in 30% of cases, persistent effects manifest into post-concussive syndrome (PCS; Lewine et al., 2007). Symptoms such as cognitive and memory impairments (Vanderploeg et al., 2005), as well as motor deficiencies (De Beaumont et al., 2007), can be long term. Pathologically, injuries can be either primary or secondary. Primary injuries occur at the time of the trauma and can include fracture, as well as an increase in pressure and bleeding. Secondary injuries occur after the traumatic event and may involve disruption in system function at the molecular and cellular level. This may be in the form of problems with neurotransmitter release and reuptake, scarring from astrocytes at the site of injury, inflammation, and necrosis and apoptosis of neuronal and glial cells (Mckee and Daneshvar, 2015).

Adult mammals have limited neuroregenerative capabilities after an injury to the central nervous system (CNS; Lieschke and Currie, 2007). Interestingly, zebrafish (*Danio rerio*) maintain the remarkable ability to regenerate and repair neural tissue throughout adulthood (März et al., 2011; Kishimoto et al., 2012; Skaggs et al., 2014). While some important mechanisms of zebrafish neuroregeneration have been identified (Kishimoto et al., 2012; Kyritsis et al., 2012), no one has examined the entire transcriptome of the adult zebrafish during neuroregeneration.

Current zebrafish TBI studies use stab or lesion models to induce injury (Kroehne et al., 2011; Kishimoto et al., 2012; Kyritsis et al., 2012; Skaggs et al., 2014). Although these models are novel for the analysis of focal injuries in the zebrafish brain, they could be considered moderate or severe models of TBI, as they both involve penetration from the epidermal layer through the blood–brain barrier (Saatman et al., 2008). Here, we adapted a novel mTBI model for zebrafish by applying modifications to an accepted rodent weight drop apparatus (Mychasiuk et al., 2014). Our model inflicts a nonpenetrating, diffuse mTBI injury that allows for the study of the entire zebrafish brain transcriptome during neuroregeneration.

The purpose of this study was twofold: first, to develop a model of mTBI using adult zebrafish; and second, to elucidate the genetic pathways of the adult zebrafish during the peak injury response and the peak of neuroregeneration. To do so, we examined differential gene expression in adult zebrafish at 3 days postinjury (dpi), which corresponded to the peak injury response and a significant deficit in spatial memory, and 21 dpi, the estimated peak neuroregeneration response, in comparison to sham controls that did not receive an mTBI. We then collected data using RNA-sequencing (RNA-seq) followed by transcriptome analysis. Each time point was compared to sham controls to identify differentially expressed genes (DEGs) and affected gene ontology (GO) clusters in biological processes, cellular components, and molecular functions.

## MATERIALS AND METHODS

### Animals

All animals used were commercially acquired adult zebrafish (*Danio rerio*), homozygous with lof^dt2^, a long-fin mutation. The fish were maintained in accordance with standard protocol on a 14-h/10-h light/dark schedule at 28.5°C, and all procedures were approved and conducted within the W.M. Keck Science Department Institutional Animal Care and Use Committee, approval number 16-832. For the RNA-seq analysis, a total of 30 fish were used and divided into three main groups: control (*n* = 10), 3 dpi (*n* = 10), and 21 dpi (*n* = 10). Each main group was then divided into two subgroups (*n* = 5) for pooled samples for RNA-seq analysis. We used 15 males and 15 females equally divided between each time point. The fish were evaluated at varying intervals of the recovery process at 3 and 21 dpi. At each interval, the fish were killed in accordance with recommended procedures of prolonged exposure to tricaine, so as not to cause unnecessary harm to the fish (Collymore et al., 2014).

### Weight drop model

**Figure 1.**
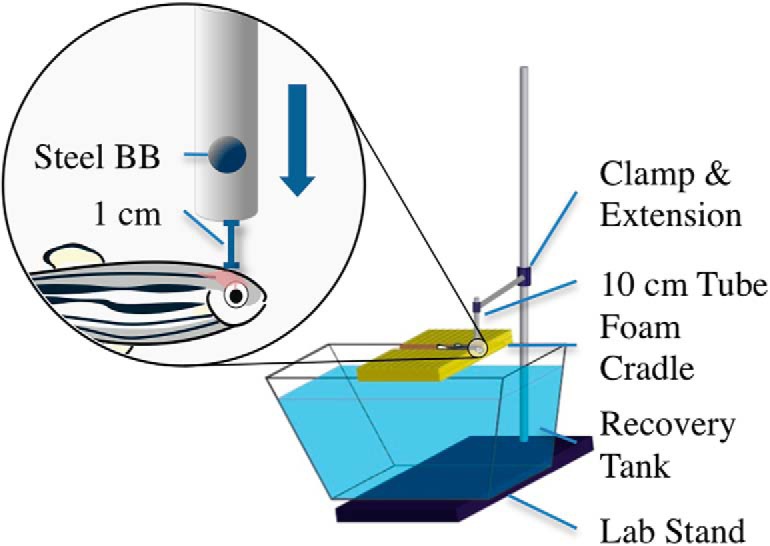
The mTBI weight-drop model for adult zebrafish. This novel model allows administration of a nonpenetrating, diffuse injury to the brain of an adult zebrafish. The fish are anesthetized in 0.02% tricaine-S (MS-222), placed on the foam cradle, and given a strike from a 0.33-g ball bearing, delivering a force of 0.0032 N and an impact energy of 35 mJ. The bottom of the guide tube is fixed ∼1 cm above the zebrafish cranium, ensuring impact in a targeted location between the eyes of the fish.

As previously mentioned, current TBI models for zebrafish administer only moderate or severe TBIs. Our novel mTBI model is a weight drop model adapted from an existing rodent mTBI model (Mychasiuk et al., 2014). The apparatus consists of a 36-inch laboratory support stand as the base, with a three-prong adjustable clamp that holds a 10-cm plastic tube with an outer diameter of 12.7 mm (0.5 inches) and an inner diameter of 4.76 mm (0.187 inches) from ePlastics. An Aquaneering M3 ZT280 2.8 L tank was filled with system water and placed on the support stand base beneath the tube. A foam block was set on top of the medium recovery tank to act as a cradle for the fish, and the tube was positioned to be ∼1 cm above the cranium of the fish. The foam, black polyethylene foam with 1.7-pound density, was cut to 15 × 7 × 1.9 cm, and then a Dremel hand-held rotary tool with a 0.75-inch steel brush was used to define the tracks 11 cm apart from each other and 0.5 cm deep (Fig. 1). The groove that stabilizes the fish during the procedure was bored using the same steel brush attachment to grind the foam, forming a 0.75-cm wide and 0.5-cm deep groove across the surface of the foam.

Fish were anesthetized in 0.02% tricaine-S (MS-222) solution, made from a 5-mL aliquot in 95 mL system water. With a large number of animals, two 150-ml beakers were used to speed the process, allowing for alternating times staggered at 60 s. Sufficient anesthesia was determined with negative motor response to a tail pinch with forceps and positive gill fluctuation. The fish were then quickly removed from the MS-222 solution and placed in the foam cradle beneath the plastic tube with its dorsal side erect, ventral side to the foam, and anterior end hanging just over the edge of the foam so that the gills were in line with the edge of the foam block. The superior side of the head was aligned below the tube by looking through the tube and ensuring a weight would strike the cranium. A single 4.5-mm steel BB, with a mass of 0.33 g and weight of 0.0032 N, was dropped, reaching a maximum speed of 1.5 m/s in 0.073 s, and striking the cranium with a maximum impact energy of 35 mJ. The fish were then quickly placed into the fish water below the foam. This process was repeated for all fish in the mTBI groups. For the control group, the fish were anesthetized with the same solution as the mTBI groups. The control fish were then placed on the foam cradle for approximately the same time the mTBI fish were placed on the foam cradle, 10 s, then placed in the recovery tank. Once recovered, the fish were placed in tanks marked as control, 3 dpi, or 21 dpi.

### Behavioral testing

To test brain function via behavior between control and mTBI zebrafish, a memory and swimming test apparatus was adapted and modified from the aquatic three-chamber arena used in an experiment to characterize and compare behavior indicative of spatial memory in zebrafish (Norton and Bally-Cuif, 2010; Barba-Escobedo and Gould, 2012; Stewart et al., 2014). For our experimentation, this three-chambered arena took the form of a Plexiglas T-maze. The middle arm served as a runway, and the two side arms were partitioned off from the runway by a transparent Plexiglas door. Fish were acclimated to the T-maze for 8 d before mTBI induction. On that day (day –8), all experimental zebrafish (*n* = 12) explored for 15 min, followed by half of the fish per session (*n* = 6) on day –7, and finally individual exploration (*n* = 1) for day –6. After acclimation to the tank, zebrafish (*n* = 12) underwent spatial memory training sessions once per day over 5 d (days –5 to –1). Because of the zebrafish preference for shoaling, free swimming, separately housed zebrafish (*n* = 5) were placed in the two side arms of the maze to serve as a shoaling reward at the end of the runway. In each training session, individual fish were placed at the beginning of the middle arm, or the runway, of the T-maze and allowed to swim freely until reaching the transparent Plexiglas partition at the end of the runway that separated the subject from the shoaling fish in the two side arms. This was done to train experimental zebrafish spatial memory regarding the location of the shoaling fish. Time to reach the transparent partition, or shoaling time, was videorecorded for each training session. The day after the final training, on day 0 of the experiment, the fish were randomly placed into 2 groups (*n* = 6), and 1 group received an mTBI. After a 1 h recovery time, time to shoal spatial memory tests were conducted on each fish in its respective group. This test was repeated daily for 3 additional dpi. In this test, all variables were identical to training except for the removal of shoaling fish from the maze. The videos were then analyzed via a blind researcher who recorded the duration in seconds of time to shoal. This behavioral test was replicated a second time (*n* = 6) to ensure validity. Repeated-measures ANOVA was used to determine significance between groups and between days and the interaction effect between both groups and days. A Tukey multiple comparisons *post hoc* test was then performed if significance was found from the initial repeated-measures ANOVA (Prism 6, GraphPad Software).

### RNA sample preparation

After mTBI, the zebrafish (*n* = 10) had a 93.3% survival rate. The remaining fish were killed, and the brain tissue was placed in RNAlater. RNA was isolated and purified from the brain tissue samples with the RNAeasy Minikit by Qiagen according to the manufacturer’s instructions. The purified RNA was pooled into two samples for each of the three groups and sequenced as unpaired, single-ended strands by GeneWiz.

### Differential gene expression analysis

RNA-sequencing FASTQ files were uploaded to Galaxy (usegalaxy.org), an open-source data analysis website equipped with bioinformatic packages and tools (Afgan et al., 2016). The sequencing files were trimmed according to a quality score ≥20 using the FASTQ Quality Trimmer, and the 6-nucleotide-long Illumina indices were trimmed off the 5′ ends of the RNA using Trim Galore!. Using Tophat, the trimmed sequences were mapped to the Genome Reference Consortium Zebrafish Build 10 (GrCz10/DanRer10) assembly of the zebrafish genome (released September 2014). The number of reads mapped to each gene feature of the reference genome was counted with Htseq-count, and differences in counts between control and mTBI groups were determined with DESeq2 (Love et al., 2014), a negative binomial distribution model. Significant DEGs were identified according to a 0.05 *p*-value corrected for a false discovery rate (FDR) for multiple testing. Statistical analysis was performed in Prism.

### Gene ontology

The Ensembl identification (uswest.ensembl.org) for each gene was determined using the Ensembl genome browser (Yates et al., 2016). The Ensembl identifiers were input into GOrilla (cbl-gorilla.cs.technion.ac.il; a customizable web source that integrates biological datasets), which grouped the significant DEGs according to three umbrella categories of cellular function: biological processes, cellular components, and molecular function (Eden et al., 2009).

### Quantitative RT-PCR

cDNA was synthesized from total RNA from three pooled fish per time point using cDNA RT Kit 4368814 (Thermo Fisher Scientific), and quantitative PCR was performed using SYBR green master mix (Life Technologies) in an ABI PRISM 7900HT (Life Technologies) instrument using the following primers: Apoeb-F, 5′-GCAGATGACGTGAAGAACCG-3′; Aboeb-R, 5′-GTTGCTACGGTGTTGCGGAT-3′, Loxl2b-F, 5′-AAGCAGGGATTTACACTTCGGA-3′, Loxl2b-R, 5′-AGCCAGCATAATGACAGAGGC-3′, Notch1b-F, 5′-GTAGATGCAGCGATGGTGTTG-3′, Notch1b-R, 5′-AGCCGTCTCGTAACTTCCTTC-3′. ΔCt was calculated using Elongation factor 1-α (EF1α) as a reference gene, using the following primers: EF1a-F, 5′-CAGCTGATCGTTGGAGTCAA-3′, and EF1a-R, 5′-TGTATGCGCTGACTTCCTTG-3′. Relative expression levels were determined using the ΔΔCt method (Livak and Schmittgen, 2001), normalized to age-matched controls that did not receive mild traumatic brain injury.

## Results

### Mild traumatic brain injury model

Modification of an existing rodent weight drop model was necessary to create a suitable fish model for mTBI (Fig. 1; Mychasiuk et al., 2014). The mTBI weight drop model for adult zebrafish establishes a protocol for consistent application of a head injury that represents a blow or a strike. Observations are that the procedure leaves little epidermal damage, with a minor number of cases presenting a small indentation from the ball bearing at the strike location. This procedure is modestly rapid from the time the fish is placed in the anesthesia, until the time it is placed in the recovery tank.

### Behavioral analysis

To validate our mTBI model, we performed a spatial memory behavioral test. This test measured the time it took fish to remember the location of a school of fish they had previously swam with. Previous studies have shown that zebrafish prefer swimming in groups, also known as shoaling, to avoid predation and improve foraging in the wild (Miller and Gerlai, 2012). This test specifically measured the animal’s spatial memory with regard to time to shoal (Barba-Escobedo and Gould, 2012; Miller and Gerlai, 2012; Stewart et al., 2014). Results from this test confirmed that mTBI fish took significantly longer to locate the correct spatial location of shoaling than sham controls on the day of mTBI induction as well as 1 and 3 dpi. Interestingly, the mTBI group demonstrated a clear improvement over in time to shoal over the 0–3 dpi, though this trend was not statistically significant. While the largest difference in both behavioral tests occurred between groups at day 0, the day of mTBI induction, clear differences between groups were still discernable at 3 dpi (Table 1).

**Table 1.** Time to shoal was found to be significantly longer in the mTBI versus control group

Sample	Pre-injury	0 dpi	1 dpi	2 dpi	3 dpi	*p*-value
Control	9.9 ± 3.949	13.405 ± 5.429*	11.223 ± 2.753*	8.535 ± 2.534	6.62 ± 3.37*	
mTBI	9.9 ± 3.949	107.488 ± 63.267*	48.378 ± 29.949*	17.82 ± 11.197	15.97 ± 6.65*	

Spatial memory testing was conducted 1 h after injury on the day of mTBI induction (day 0) as well as once per day for 3 d after the administration of mTBI and sham injuries. Average time to shoal measured in seconds for mTBI and control zebrafish found a statically significant difference between mTBI and sham controls, although no significant effect was found between days or among the interaction effect, *p* = 0.034, *F*(1,5) = 8.324. Tukey’s multiple comparison *post hoc* test confirmed that days 0, 1, and 3 dpi were significantly different between groups (A). *n* = 6 per group.

*Significant difference between mTBI and control groups on that specific day by Tukey multiple comparison *post hoc* analysis (*p* < 0.05).

### RNA analysis

**Figure 2.**
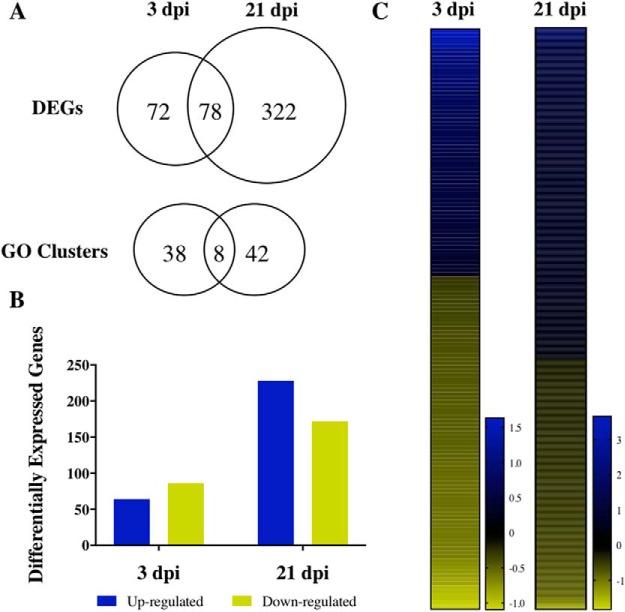
Summary of differential gene analysis and GO enrichment results. The number of shared and mutually exclusive DEGs (***A***) and GO categories between 3 and 21 dpi (*p* < 0.05). The total number of up and down-regulated genes (***B***) and heat maps (***C***) showing log-2 fold change of gene expression in comparison to the control group.

To assess changes in gene expression in the brain following mTBI, control, 3-dpi, and 21-dpi animals were killed and had their brains prepared for RNA-seq analysis. The RNA samples were sequenced by next-generation sequencing, which, on average, generated more than 27 million reads per sample (Table 2). Filtered and trimmed reads were then mapped to the zebrafish DanRer10 reference genome with an average mapping rate of 91.5%. Exon features of mapped reads from a non–strand-specific assay for 3 and 21 dpi were counted with Htseq-count in union mode and compared to control read counts using DESeq2 with a parametric fit type. The significant DEGs were identified according to an FDR-corrected *p*-value of 0.05, resulting in 150 DEGs at 3 dpi and 400 DEGs at 21 dpi (Fig. 2*A*; Tables 5, 6, and 7). At 3 dpi, 43% of DEGs were up-regulated, in comparison to 57% of DEGs at 21 dpi (Fig. 2*B*). The log-2 fold change range for DEGs at 21 dpi was nearly 5.5, which was twice the log-2 fold change range observed at 3 dpi (Fig. 2*C*).

**Table 2.** Alignment results of RNA sequence samples to zebrafish reference genome

Sample	Raw reads	Filtered reads	Mapped	Mapping rate (%)
Control A	25,848,096	25,843,032	23,655,092	91.50
Control B	27,542,279	27,537,682	25,250,525	91.70
3 dpi A	27,655,407	27,650,295	25,271,599	91.50
3 dpi B	29,271,785	29,265,732	26,784,112	91.40
21 dpi A	26,258,485	26,253,619	24,029,275	91.40
21 dpi B	26,700,791	26,696,129	24,409,763	91.50

**Figure 3.**
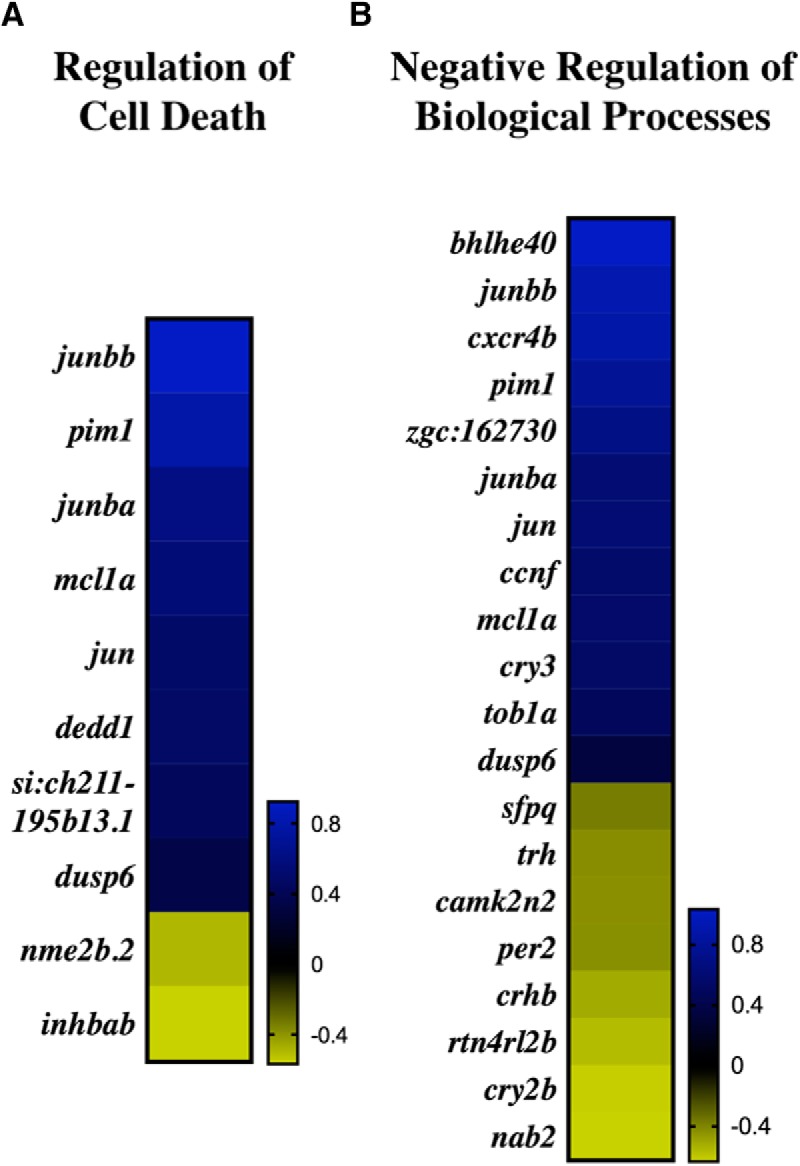
GO categories encompassing early response to injury at 3 dpi. Log-2 fold change of DEGs within regulation of cell death (***A***) and negative regulation of biological processes (***B***).

**Figure 4.**
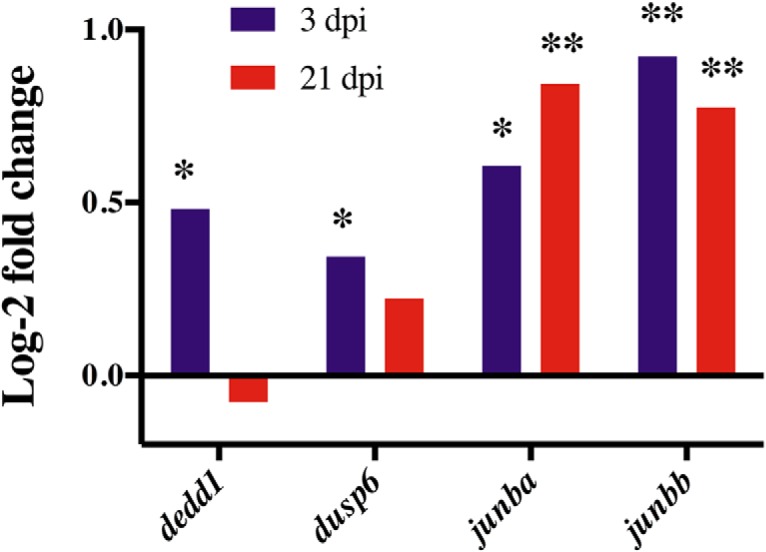
Expression of pro-apoptotic DEGs at 3 and 21 dpi. At 3 dpi, *dedd1* (*p* = 0.0207), *dusp6* (*p* = 0.0239), *junba* (*p* = 0.0008), and *junbb* (*p* < 0.0001) were significantly up-regulated. Only *junba* and *junbb* were significantly upregulated at 21 dpi (*p* < 0.0001). * *p* < 0.05; ** *p* < 0.0001.

The DEGs were then sorted according to their respective GO categories at an FDR-corrected 95% confidence interval for a total of 46 and 50 GO terms at 3 and 21 dpi, respectively (Fig. 2*A*). At 3 dpi, 60% of the GO categories were enriched within biological processes, 40% within molecular function, and none within cellular components (Table 3). At 21 dpi, there were GO clusters associated with each category with 50% in biological processes, 20% in molecular function, and 30% in cellular components. Between 3 and 21 dpi, 11% of the biological processes GO categories and 7% of the molecular function GO categories were shared. At 3 dpi, GO clusters mainly encapsulated chemical and hormonal signaling pathways, including the cyclic adenosine monophosphate (cAMP) and mitogen-activated protein (MAP) kinase pathways (Table 3). In addition, more than 30% of the GO categories related to phosphate-containing compounds, dephosphorylation, and phosphatase activity. Two notable GO clusters include regulation of cell death and negative regulation of biological processes, which exhibited an average log-2 fold change of 0.36 across 10 genes and 0.20 across 20 genes, respectively (Fig. 3). Specific to the regulation of cell death GO category, death effector domain (*dedd1*) promotes apoptosis (Lee et al., 2000) and inhibits proliferation (Arai et al., 2007). The regulation of *dedd1* was significant at 3 dpi (*p* = 0.0207; Fig. 4), where it was up-regulated 35% more than the average regulation of DEGs comprising regulation of cell death (Fig. 3*A*). Between the two GO clusters, 6 DEGs were shared, including dual-specificity phosphatase 6, *dusp6*, which promotes p53-mediated cell death (Piya et al., 2012), and was significantly upregulated at 3 dpi (*p* = 0.0239, Fig. 4). Additionally, *junba* and *junbb*, two orthologs of the mammalian *Junb* gene found to be required for tissue regeneration in zebrafish (Ishida et al., 2010), were significantly upregulated at both 3 dpi (*p* = 0.0008 and *p* < 0.0001) and 21 dpi (*p* < 0.0001; Fig. 4).

**Table 3.** Representative sample of GO categories associated with DEGs at 3 dpi

Category	GO ID	Description	*p*-value
Biological process	GO:0051591	Response to cAMP	1.09 × 10^5^
Biological process	GO:0006470	Protein dephosphorylation	3.85 × 10^5^
Biological process	GO:0048519	Negative regulation of biological process	5.77 × 10^4^
Biological process	GO:0010941	Regulation of cell death	8.35 × 10^4^
Molecular function	GO:0017017	MAP kinase tyrosine/serine/threonine phosphatase activity	1.86 × 10^5^
Molecular function	GO:0005184	Neuropeptide hormone activity	4.60 × 10^4^

**Figure 5.**
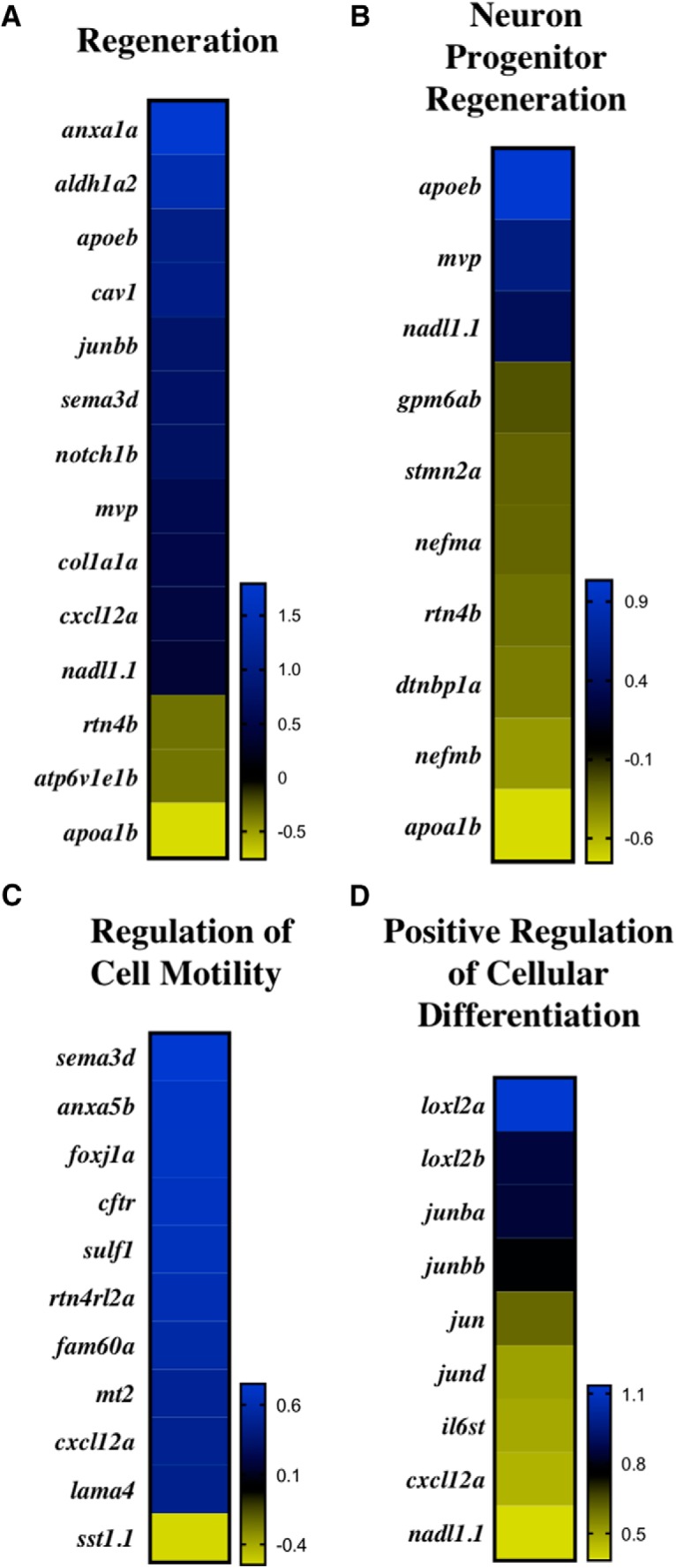
Regeneration GO categories at 21 dpi. Log-2 fold change of DEGs within regeneration (***A***), neuron progenitor regeneration (***B***), regulation of cell motility (***C***), and positive regulation of cellular differentiation (***D***).

**Figure 6.**
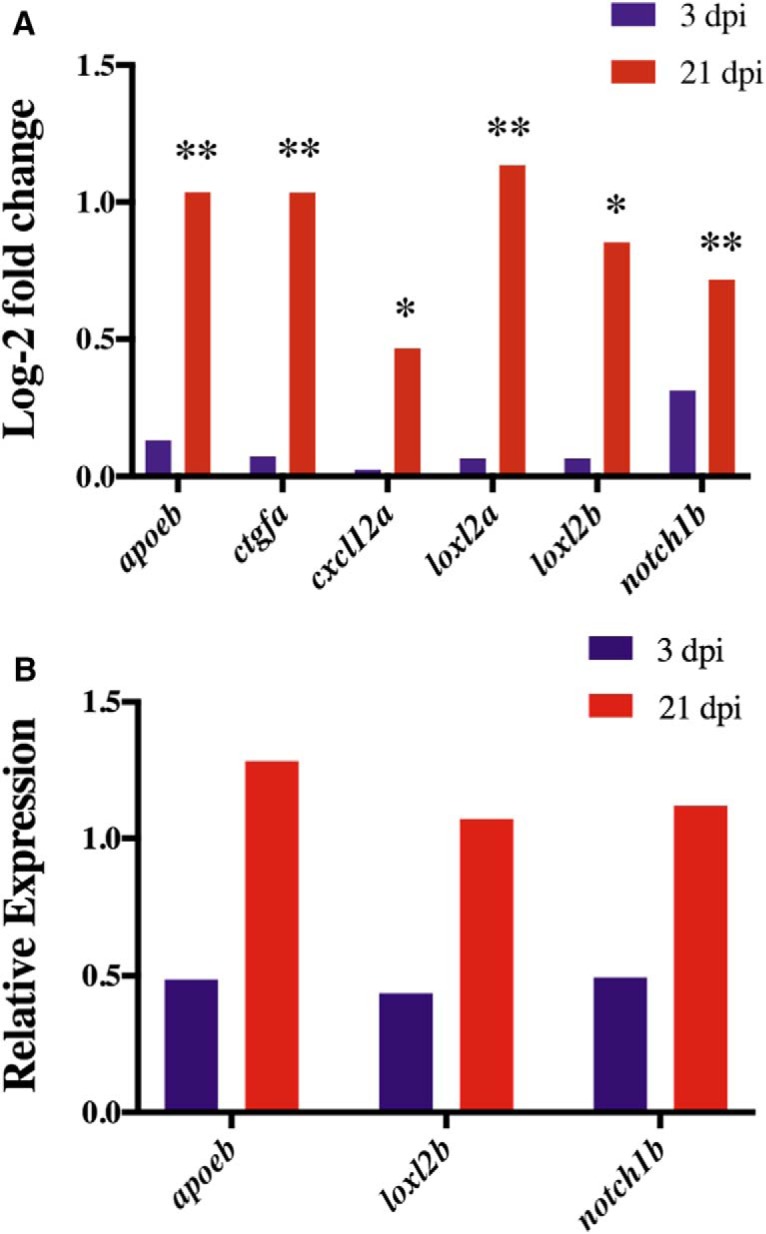
Expression of DEGs within neuroregeneration GO categories at 3 and 21 dpi. At 21 dpi, *cxcl12a* (*p* = 0.0095) and *loxl2b* (*p* = 0.0004) were significantly upregulated (***A***). The neuroregeneration DEGs *apoeb*, *ctgfa*, *loxl2a*, and *notch1b* were also significantly upregulated at 21 dpi (*p* < 0.0001); ***A***). * *p* < 0.05; ** *p* < 0.0001. qPCR shows that *apoeb*, *loxl2b*, and *notch1b* were downregulated at 3dpi but expression increased at 21 dpi (***B***).

In comparison to the enriched GO clusters at 3 dpi, which largely comprised negatively regulated pathways, GO clusters at 21 dpi were characterized by positively regulated pathways involved in neural repair, neuroregeneration, and development. Specifically, 24% of the biological processes GO categories at 21 dpi were developmental, and 16% involved regeneration. An additional 20% of the molecular function and 27% of the cellular component GO categories involved ATP pathways (Table 4). Four GO clusters encompassing neural repair and neuroregeneration DEGs at 21 dpi were neuroregeneration, neuron progenitor regeneration, regulation of cell motility, and positive regulation of cellular differentiation (Fig. 5). Within regeneration, nearly 79% of the DEGs were significantly up-regulated, with an average log-2 fold change of 0.57. Required for neurogenesis in both mammals (Wang et al., 2009) and zebrafish (Kishimoto et al., 2012), *notch1b* was significantly up-regulated at 3 dpi (*p* = 0.026) and 21 dpi (*p* < 0.0001, Fig. 6*A*). In contrast to the regeneration GO cluster, only 30% of the DEGs within neuron progenitor regeneration were up-regulated (Fig. 5*B*). Categorized into both the regeneration and neuron progenitor regeneration GO clusters, a gene involved in amyloid beta clearance, *apoeb* (Castellano et al., 2011), was significantly up-regulated at 21 dpi (*p* < 0.0001; Fig. 6*A*,*B*).

**Table 4.** Neuroregeneration GO categories of interest associated with significant DEGs at 21 dpi

Category	GO ID	Description	*p*-value
Biological process	GO:0031175	Neuron projection development	2.56 × 10^6^
	GO:0031099	Regeneration	3.30 × 10^6^
	GO:0032502	Developmental process	1.19 × 10^4^
	GO:0031102	Neuron projection regeneration	1.57 × 10^4^
	GO:2000145	Regulation of cell motility	2.63 × 10^4^
	GO:0045597	Positive regulation of cell differentiation	4.54 × 10^4^
	GO:0051094	Positive regulation of developmental process	4.54 × 10^4^
	GO:0032332	Positive regulation of chondrocyte differentiation	8.18 × 10^4^
Molecular function	GO:0042626	ATPase activity, coupled to transmembrane movement of substances	1.83 × 10^4^
Cellular component	GO:0005882	Intermediate filament	5.81 × 10^4^

**Table 5.** DEGs unique to 3 dpi

Gene	Log2 fold change (*p*–value)
nupr1	1.05 (6.22 × 10^10^)
nr1d2b	0.94 (6.06 × 10^12^)
per1b	0.91 (5.68 × 10^10^)
cxcr4b	0.88 (5.90 × 10^6^)
tagapb	0.78 (3.48 × 10^5^)
pfkfb3	0.71 (9.21 × 10^6^)
p4ha1b	0.67 (0.0036)
dusp2	0.65 (0.0044)
phgdh	0.64 (0.0005)
mknk2a	0.62 (0.0072)
s1pr5a	0.62 (0.0039)
zgc:194659	0.56 (0.0296)
ccnf	0.56 (0.0245)
mcl1a	0.56 (0.0003)
cry3	0.54 (0.0136)
ier5	0.53 (0.0005)
znf395b	0.53 (0.0173)
ccr9a	0.52 (0.0429)
elac2	0.51 (0.0183)
nfkbiab	0.49 (0.0044)
tob1a	0.49 (0.0058)
dedd1	0.48 (0.0207)
ctps1b	0.45 (0.0424)
glipr2l	0.45 (0.0311)
pdcd4a	0.44 (0.0054)
ak4	0.43 (0.0373)
dars	0.39 (0.0362)
arhgef9b	0.38 (0.0119)
slc7a8a	0.38 (0.0424)
dusp6	0.34 (0.0239)
ewsr1a	–0.29 (0.0424)
slc25a12	–0.35 (0.0423)
cirbpa	–0.35 (0.0164)
alas1	–0.36 (0.0142)
jph3	–0.37 (0.0454)
vim	–0.40 (0.0239)
ptp4a2b	–0.40 (0.0025)
hspa8	–0.40 (0.0016)
atf7ip	–0.41 (0.0069)
impdh1b	–0.41 (0.0103)
trh	–0.43 (0.0259)
camk2n2	–0.43 (0.0038)
susd6	–0.43 (0.0080)
npb	–0.44 (0.0132)
slc6a17	–0.45 (0.0046)
atad3b	–0.45 (0.0239)
g3bp1	–0.46 (0.0006)
cdk5r2b	–0.46 (0.0424)
cad	–0.46 (0.0044)
pcdh1g31	–0.46 (0.0424)
gpt2l	–0.47 (0.0244)
pcsk1	–0.48 (0.0021)
crhbp	–0.51 (0.0004)
mri1	–0.53 (0.0191)
rorcb	–0.54 (0.0102)
rbm12	–0.55 (0.0041)
cyp39a1	–0.56 (0.0046)
si:ch73-52e5.2	–0.57 (0.0048)
dnajc9	–0.57 (0.0016)
rtn4rl2b	–0.57 (0.0102)
plxnb2b	–0.59 (0.0004)
pdxkb	–0.60 (0.0073)

hspa4a	–0.62 (5.90 × 10^6^)
cry2b	–0.63 (4.44 × 10^8^)
nab2	–0.63 (0.0054)
clocka	–0.64 (0.0017)
hmox1a	–0.70 (0.0018)
LOC564685	–0.72 (0.0004)
ppm1e	–0.78 (7.94 × 10^8^)
pde10a	–0.79 (3.69 × 10^6^)
arntl2	–0.93 (1.45 × 10^6^)
nr1d4b	–1.05 (7.88 × 10^9^)

**Table 6.** DEGs unique to 21 dpi

Gene	Log2 fold change (*p*–value)
postnb	3.66 (4.83 × 10^128^)
fn1a	2.06 (1.26 × 10^37^)
wu:fj16a03	1.94 (9.95 × 10^41^)
anxa1a	1.79 (3.14 × 10^25^)
s100a10b	1.64 (1.63 × 10^31^)
serpinf1	1.63 (6.26 × 10^19^)
tfpia	1.57 (4.67 × 10^22^)
col1a2	1.46 (3.10 × 10^15^)
aldh1a2	1.43 (4.14 × 10^21^)
alpi.1	1.31 (4.03 × 10^10^)
fn1b	1.21 (4.58 × 10^11^)
si:ch211-69i14.4	1.14 (1.72 × 10^11^)
loxl2a	1.13 (1.89 × 10^7^)
ptrfb	1.12 (5.61 × 10^8^)
cldn11a	1.10 (4.74 × 10^11^)
pde6h	1.09 (2.18 × 10^7^)
slc2a12	1.05 (8.14 × 10^8^)
sfrp1a	1.05 (1.91 × 10^7^)
tgm2b	1.05 (2.93 × 10^7^)
slc16a9a	1.04 (2.17 × 10^9^)
apoeb	1.04 (1.09 × 10^11^)
rpe65a	1.03 (4.06 × 10^6^)
ctgfa	1.03 (7.82 × 10^10^)
adm2a	1.03 (1.87 × 10^6^)
cav1	1.03 (2.47 × 10^8^)
mgp	1.01 (3.04 × 10^6^)
anxa2a	1.01 (6.31 × 10^11^)
capn2a	1.00 (5.34 × 10^6^)
ckba	0.99 (1.18 × 10^7^)
ndrg1a	0.98 (5.64 × 10^7^)
slc1a5	0.97 (3.24 × 10^7^)
bhmt	0.96 (4.82 × 10^14^)
h1fx	0.96 (1.53 × 10^14^)
nmrk2	0.96 (2.65 × 10^5^)
icn	0.94 (9.19 × 10^6^)
si:ch211-80h18.1	0.93 (1.13 × 10^5^)
tes	0.92 (7.60 × 10^6^)
slc13a5a	0.92 (4.67 × 10^6^)
lamb1b	0.92 (1.18 × 10^7^)
steap4	0.91 (3.71 × 10^5^)
si:dkey-239i20.4	0.90 (9.92 × 10^6^)
ehd2b	0.90 (6.72 × 10^6^)
tspan36	0.90 (4.34 × 10^5^)
slc13a1	0.89 (0.0001)
asmt	0.89 (0.0001)
zgc:114041	0.88 (0.0002)
irbp	0.86 (0.0002)
loxl2b	0.85 (0.0004)
cldn7a	0.83 (7.35 × 10^5^)
sdpra	0.82 (0.0007)
olfml3a	0.82 (0.0006)
atp1b1a	0.81 (3.90 × 10^8^)
krt4	0.81 (0.0008)
arrdc3b	0.81 (1.58 × 10^5^)
ccdc40	0.81 (0.0005)
fam65c	0.80 (0.0011)
col5a2a	0.80 (2.25 × 10^6^)
mxra8b	0.80 (0.0011)
hhla2a.1	0.80 (0.0010)
socs3b	0.79 (0.0012)
zgc:77517	0.78 (6.06 × 10^5^)
f11r.1	0.78 (0.0018)

dkk3b	0.78 (0.0018)
ctnna1	0.77 (2.27 × 10^5^)
krt8	0.76 (8.73 × 10^10^)
urp2	0.76 (0.0017)
ca5a	0.76 (7.15 × 10^8^)
pde6g	0.75 (8.21 × 10^5^)
pmela	0.75 (0.0010)
sema3d	0.75 (0.0032)
zgc:174895	0.75 (0.0031)
aldh4a1	0.74 (4.37 × 10^5^)
sfrp5	0.74 (0.0011)
gfap	0.74 (5.08 × 10^10^)
fabp11b	0.73 (0.0002)
znf395a	0.73 (0.0012)
zgc:158423	0.73 (7.28 × 10^8^)
itgbl1	0.72 (0.0045)
notch1b	0.72 (2.09 × 10^5^)
abat	0.71 (5.40 × 10^9^)
cpt1b	0.71 (0.0030)
olfml3b	0.70 (0.0051)
fabp7b	0.70 (0.0053)
slc7a5	0.70 (0.0001)
anxa5b	0.69 (0.0081)
tsku	0.69 (0.0006)
col1a1b	0.69 (0.0049)
aldoab	0.69 (0.0084)
zgc:113263	0.69 (0.0095)
foxj1a	0.69 (0.0088)
plod2	0.69 (0.0098)
msrb2	0.68 (4.03 × 10^5^)
pkd2	0.68 (0.0013)
sdprb	0.68 (0.0036)
slc16a9b	0.68 (0.0032)
zgc:73075	0.68 (0.0111)
si:dkey-184p18.2	0.68 (0.0113)
foxc1b	0.68 (0.0016)
cftr	0.67 (0.0062)
anxa11a	0.67 (0.0011)
afap1l1a	0.66 (0.0150)
stra6	0.66 (0.0125)
zgc:85866	0.66 (0.0150)
rspo3	0.66 (0.0134)
gpcpd1	0.66 (3.35 × 10^5^)
ltbp3	0.65 (0.0012)
sulf1	0.65 (0.0067)
nccrp1	0.65 (0.0011)
serpine1	0.64 (0.0135)
ftr82	0.63 (0.0058)
ctsk	0.63 (0.0235)
s100v2	0.63 (0.0007)
pcolcea	0.63 (0.0228)
rhoub	0.62 (0.0256)
wu:fc46h12	0.62 (0.0138)
rtn4rl2a	0.61 (8.68 × 10^8^)
qsox1	0.61 (0.0315)
tyrp1b	0.61 (0.0155)
arr3a	0.61 (0.0243)
rbp4l	0.61 (0.0229)
abcb4	0.61 (0.0030)
tspo	0.61 (0.0197)
si:ch211-165i18.2	0.60 (0.0116)
gb:eh507706	0.60 (0.0370)

fxyd1	0.60 (4.17 × 10^5^)
pde6c	0.59 (0.0413)
cd99	0.59 (0.0024)
dhrs3a	0.59 (0.0370)
b3gnt7	0.59 (0.0425)
egr2b	0.59 (0.0267)
snx16	0.59 (0.0004)
hsd11b2	0.59 (0.0096)
acta2	0.58 (0.0024)
add3a	0.58 (2.66 × 10^5^)
cthrc1a	0.58 (0.0152)
gstt1b	0.58 (0.0315)
lxn	0.57 (0.0117)
cyp2ad3	0.57 (0.0132)
mvp	0.57 (0.0131)
alpl	0.56 (0.0190)
tagln	0.56 (0.0415)
opn1lw2	0.56 (0.0421)
glud1a	0.56 (0.0161)
igfbp5b	0.56 (0.0001)
fam60a	0.56 (0.0399)
mgea5	0.56 (4.02 × 10^5^)
lamc1	0.56 (0.0031)
serpinh1a	0.55 (0.0385)
htra1b	0.54 (0.0102)
ifngr1	0.54 (0.0229)
npr3	0.54 (0.0250)
rrad	0.53 (0.0274)
colec12	0.53 (0.0184)
plxdc2	0.53 (0.0236)
twsg1a	0.53 (0.0248)
gldc	0.52 (0.0017)
col1a1a	0.52 (0.0187)
ctsc	0.51 (0.0471)
herc3	0.51 (0.0036)
myh11a	0.51 (0.0107)
smox	0.50 (0.0013)
jund	0.50 (0.0162)
il6st	0.49 (0.0173)
myl9b	0.49 (0.0198)
mt2	0.49 (0.0276)
lpl	0.48 (0.0352)
zgc:92630	0.48 (0.0264)
pdcd4b	0.48 (0.0011)
ehd1a	0.48 (0.0495)
aoc2	0.48 (0.0017)
rorca	0.47 (0.0412)
cxcl12a	0.47 (0.0095)
lbr	0.46 (0.0498)
nucb2a	0.45 (0.0008)
lama4	0.45 (0.0135)
tln1	0.45 (0.0257)
hbaa1	0.44 (0.0228)
zgc:123105	0.44 (0.0018)
fosl2	0.43 (0.0191)
cast	0.43 (0.0470)
clu	0.42 (4.29 × 10^5^)
zfp36l2	0.42 (0.0117)
rbp4	0.39 (0.0259)
trim71	0.39 (0.0458)
nadl1.1	0.38 (0.0166)
nat8l	0.38 (0.0211)

ppdpfb	0.37 (0.0199)
p4hb	0.36 (0.0368)
rorab	0.34 (0.0297)
cpne1	0.34 (0.0472)
zgc:55733	0.31 (0.0315)
atp1a1a.1	0.28 (0.0228)
eef1a1b	0.27 (0.0432)
si:dkey-4p15.3	0.27 (0.0388)
gpm6ab	–0.24 (0.0257)
gabrb2	–0.25 (0.0448)
snap25a	–0.26 (0.0106)
snap25b	–0.26 (0.0381)
ndrg3a	–0.26 (0.0126)
cplx2l	–0.26 (0.0447)
atp6v1g1	–0.27 (0.0278)
gad1b	–0.28 (0.0176)
cdk5r2a	–0.28 (0.0145)
grin1b	–0.29 (0.0209)
stmn2a	–0.29 (0.0202)
mafba	–0.29 (0.0474)
si:ch211-251b21.1	–0.30 (0.0105)
map2k1	–0.30 (0.0240)
nefma	–0.30 (0.0225)
atp1b3b	–0.30 (0.0117)
islr2	–0.31 (0.0165)
atp6v1aa	–0.31 (0.0080)
agap2	–0.32 (0.0324)
ivns1abpa	–0.32 (0.0026)
chn1	–0.32 (0.0273)
atpv0e2	–0.33 (0.0279)
oxr1b	–0.33 (0.0248)
cp	–0.33 (0.0264)
etv5a	–0.33 (0.0485)
sypb	–0.33 (0.0095)
hexim1	–0.33 (0.0339)
zgc:65894	–0.33 (0.0474)
necap1	–0.33 (0.0069)
dpysl5a	–0.33 (0.0173)
cox8a	–0.33 (0.0231)
trim9	–0.34 (0.0065)
scg2b	–0.34 (0.0003)
chgb	–0.34 (0.0482)
diras1a	–0.34 (0.0022)
prkcda	–0.34 (0.0167)
max	–0.34 (0.0138)
cbln12	–0.34 (0.0027)
taf15	–0.34 (0.0170)
fez1	–0.34 (0.0066)
atp6v1e1b	–0.35 (0.0085)
marcksb	–0.35 (0.0064)
tuba2	–0.35 (0.0302)
atp1b1b	–0.35 (0.0061)
nptna	–0.36 (0.0025)
slc2a1a	–0.36 (0.0316)
prickle2b	–0.37 (0.0190)
syngr3b	–0.37 (0.0041)
dlg1	–0.37 (0.0010)
sltm	–0.38 (0.0053)
olfm1a	–0.38 (0.0095)
dtnbp1a	–0.38 (0.0162)
luc7l	–0.38 (0.0143)
chac1	–0.39 (6.62 × 10^5^)

kctd12.2	–0.39 (0.0125)
adcy8	–0.39 (0.0216)
b3gat2	–0.39 (0.0135)
sub1b	–0.39 (0.0235)
h2afvb	–0.39 (0.0250)
zgc:101840	–0.39 (0.0293)
stxbp6l	–0.40 (0.0008)
syt12	–0.41 (0.0010)
ifrd1	–0.41 (0.0084)
sst3	–0.41 (0.0078)
si:ch211-203b8.6	–0.42 (0.0009)
gnb5b	–0.42 (0.0285)
smyd2a	–0.42 (0.0014)
txndc12	–0.42 (0.0320)
cd9b	–0.43 (0.0093)
tubb2b	–0.43 (0.0150)
atp1b3a	–0.44 (0.0044)
ccdc85al	–0.45 (0.0095)
tiparp	–0.45 (0.0308)
hsbp1a	–0.45 (0.0402)
sumo2b	–0.45 (0.0028)
sult2st3	–0.45 (0.0081)
zwi	–0.46 (0.0150)
abcc5	–0.46 (0.0066)
stk25a	–0.46 (0.0344)
ucn3l	–0.46 (0.0171)
lmo2	–0.46 (0.0486)
oaz1b	–0.47 (1.41 × 10^5^)
phkg1a	–0.48 (0.0487)
nefmb	–0.49 (0.0071)
sox7	–0.49 (0.0349)
gng13b	–0.49 (6.36 × 10^5^)
nrn1a	–0.49 (0.0037)
csrp1b	–0.50 (0.0036)
tmbim4	–0.50 (0.0001)
snapc5	–0.50 (0.0010)
zgc:77056	–0.51 (0.0228)
hmgb3a	–0.52 (8.89 × 10^6^)
pkn1b	–0.53 (7.33 × 10^5^)
plp1a	–0.53(0.0245)
pltp	–0.53 (0.0055)
zgc:73226	–0.55 (0.0206)
mid1ip1b	–0.55 (0.0018)
rbmx	–0.56 (6.82 × 10^7^)
gapdh	–0.57 (0.0493)
si:dkey-222p3.1	–0.57 (0.0419)
siglec15l	–0.59 (0.0399)
cx27.5	–0.59 (0.0007)
oxt	–0.59 (0.0014)
slc25a22	–0.61 (0.0031)
iqch	–0.61 (0.0165)
tnnc2	–0.61 (0.0248)
cldnk	–0.62 (2.63 × 10^6^)
rnf144ab	–0.62 (0.0001)
erf	–0.66 (0.0017)
flj13639	–0.66 (1.47 × 10^7^)
mpz	–0.68 (9.11 × 10^9^)
lancl1	–0.69 (1.92 × 10^8^)
pvalb3	–0.70 (0.0007)
sult2st1	–0.71 (0.0062)
si:ch211-147k10.6	–0.71 (6.34 × 10^5^)

foxh1	–0.73 (0.0023)
apoa1b	–0.76 (0.0026)
tfap2c	–0.76 (0.0017)
olfm2b	–0.77 (4.58 × 10^11^)
mstna	–0.79 (0.0014)
asb15b	–0.80 (0.0010)
plp1b	–0.80 (9.36 × 10^9^)
tnnt3b	–0.87 (0.0001)
pth2	–0.87 (9.47 × 10^6^)
myhc4	–0.95 (4.35 × 10^6^)
mylpfa	–1.09 (5.90 × 10^7^)
atp2a1l	–1.16 (1.92 × 10^8^)
pvalb4	–1.25 (1.02 × 10^9^)
gpx1a	–1.31 (5.71 × 10^18^)

**Table 7.** DEGs shared between both 3 and 21 dpi

3 DPI		21 DPI	
Gene	Log2 fold change (*p*–value)	Gene	Log2 fold change (*p*–value)
abce1	–0.45 (0.0008)	abce1	–0.35 (0.0087)
abcf2a	–0.44 (0.0137)	abcf2a	–0.66 (5.19 × 10^7^)
acta2	0.47 (0.0459)	acta2	0.58 (0.0024)
actc1b	–0.98 (3.82 × 10^7^)	actc1b	–1.38 (2.56 × 10^11^)
adcyap1b	–0.31 (0.0233)	adcyap1b	–0.41 (2.49 × 10^5^)
arfgap1	–0.48 (0.0306)	arfgap1	–0.46 (0.0288)
atf3	0.64 (0.0044)	atf3	0.74 (0.0016)
atp1a3b	–0.36 (0.0302)	atp1a3b	–0.37 (0.0069)
barhl1b	–0.48 (0.0069)	barhl1b	–0.42 (0.0246)
bhlhe40	1.03 (1.14 × 10^22^)	bhlhe40	0.60 (1.89 × 10^7^)
btg1	0.45 (0.0007)	btg1	0.46 (0.0001)
btg2	1.42 (2.29 × 10^34^)	btg2	1.09 (8.01 × 10^14^)
cacnb4b	–0.43 (0.0012)	cacnb4b	–0.55 (1.20 × 10^7^)
ckma	–0.76 (8.73 × 10^5^)	ckma	–0.75 (0.0027)
ckmb	–1.09 (4.73 × 10^9^)	ckmb	–1.83 (2.76 × 10^20^)
cmklr1	0.77 (0.0002)	cmklr1	0.61 (0.0278)
crhb	–0.51 (0.0033)	crhb	–0.53 (0.0011)
ctdsp2	0.54 (0.0004)	ctdsp2	0.42 (0.0063)
cx44.2	0.55 (0.0277)	cx44.2	0.71 (0.0019)
ddx5	–0.49 (4.01 × 10^5^)	ddx5	–0.45 (1.34 × 10^5^)
dusp1	0.52 (0.0359)	dusp1	0.66 (0.0042)
dusp5	0.66 (1.53 × 10^6^)	dusp5	0.57 (0.0004)
efhd1	–0.42 (0.0041)	efhd1	–0.35 (0.0219)
egr1	1.10 (2.68 × 10^24^)	egr1	0.96 (1.83 × 10^15^)
eif4e1c	–0.47 (0.0049)	eif4e1c	–0.43 (0.0112)
fam49a	–0.36 (0.0072)	fam49a	–0.44 (2.34 × 10^5^)
fosab	1.63 (7.60 × 10^34^)	fosab	1.10 (2.97 × 10^11^)
gadd45ba	0.68 (3.08 × 10^5^)	gadd45ba	0.46 (0.0246)
ggctb	–0.32 (0.0291)	ggctb	–0.71 (2.72 × 10^12^)
glipr1b	–0.84 (4.00 × 10^8^)	glipr1b	–0.89 (1.02 × 10^8^)
gpr186	0.75 (9.12 × 10^5^)	gpr186	0.57 (0.0050)
higd1a	–0.45 (0.0072)	higd1a	–0.56 (1.41 × 10^5^)
histh1l	0.40 (0.0080)	histh1l	1.29 (2.75 × 10^33^)
hivep2a	–0.62 (4.00 × 10^8^)	hivep2a	–0.67 (5.08 × 10^10^)
hsd17b12a	–0.59 (0.0004)	hsd17b12a	–0.65 (7.95 × 10^5^)
ier2	0.90 (4.13 × 10^7^)	ier2	0.79 (6.26 × 10^5^)
inhbab	–0.57 (0.0284)	inhbab	–0.57 (0.0415)
jun	0.49 (0.0008)	jun	0.60 (1.22 × 10^6^)
junba	0.61 (0.0008)	junba	0.84 (1.33 × 10^7^)
junbb	0.92 (2.94 × 10^16^)	junbb	0.78 (2.56 × 10^11^)
klf13	–0.59 (0.0040)	klf13	–0.80 (0.0001)
mknk2b	0.61 (0.0004)	mknk2b	0.59 (0.0001)
nme2b.2	–0.49 (0.0467)	nme2b.2	–0.96 (1.17 × 10^6^)
npas2	–0.99 (8.77 × 10^10^)	npas2	–0.49 (0.0429)
npas4a	0.77 (0.0003)	npas4a	0.94 (1.81 × 10^12^)
npy	–0.55 (0.0006)	npy	–0.53 (7.86 × 10^5^)
nr1d4a	–0.94 (4.44 × 10^8^)	nr1d4a	–0.66 (0.0010)
nr4a1	1.34 (1.26 × 10^18^)	nr4a1	1.24 (3.28 × 10^13^)
nrsn1	–0.49 (0.0004)	nrsn1	–0.38 (0.0033)
nt5c2l1	–0.59 (0.0040)	nt5c2l1	–0.83 (3.04 × 10^5^)
odc1	–0.59 (0.0004)	odc1	0.48 (0.0030)
pcdh1g33	–0.57 (0.0110)	pcdh1g33	–0.50 (0.0068)
per2	–0.44 (0.0007)	per2	–0.42 (0.0003)
pim1	0.78 (1.56 × 10^8^)	pim1	0.53 (1.97 × 10^6^)
plk2b	0.56 (0.0033)	plk2b	0.56 (0.0019)
ptp4a3	–0.54 (0.0007)	ptp4a3	–0.59 (9.80 × 10^5^)
ptprna	–0.40 (0.0013)	ptprna	–0.43 (5.86 × 10^5^)
rcan2	–0.38 (0.0394)	rcan2	–0.40 (0.0166)
rcan3	–0.34 (0.0093)	rcan3	–0.28 (0.0445)
rtn4b	–0.36 (0.0136)	rtn4b	–0.34 (0.0250)
sfpq	–0.38 (0.0352)	sfpq	–0.38 (0.0190)

sgsm3	–0.34 (0.0454)	sgsm3	–0.34 (0.0188)
si:ch211-105j21.7	0.91 (5.37 × 10^6^)	si:ch211-105j21.7	0.63 (0.0231)
si:ch211-195b13.1	0.44 (0.0472)	si:ch211-195b13.1	0.42 (0.0039)
si:ch211-237l4.6	–0.51 (0.0135)	si:ch211-237l4.6	–0.49 (0.0188)
si:dkey-238o13.4	–0.83 (2.18 × 10^10^)	si:dkey-238o13.4	–0.85 (1.95 × 10^13^)
sik1	0.96 (4.85 × 10^11^)	sik1	0.94 (2.31 × 10^9^)
slc4a2b	0.54 (0.0351)	slc4a2b	1.65 (1.71 × 10^21^)
spred3	–0.50 (0.0002)	spred3	–0.50 (2.87 × 10^5^)
srsf5b	0.40 (0.0236)	srsf5b	0.53 (1.69 × 10^5^)
sst1.1	–0.60 (0.0001)	sst1.1	–0.55 (0.0011)
syt13	–0.60 (0.0002)	syt13	–0.62 (4.22 × 10^6^)
tmem198b	–0.56 (4.20 × 10^5^)	tmem198b	–0.73 (7.90 × 10^9^)
txnipa	0.42 (0.0015)	txnipa	0.77 (7.54 × 10^11^)
zgc:110340	0.57 (0.0021)	zgc:110340	0.33 (0.0196)
zgc:122979	1.51 (2.03 × 10^29^)	zgc:122979	0.95 (4.57 × 10^17^)
zgc:162730	0.70 (2.39 × 10^5^)	zgc:162730	0.68 (0.0036)
zgc:175128	1.41 (9.66 × 10^25^)	zgc:175128	1.06 (6.48 × 10^10^)

Radial glial cells (RGCs), which function as neuronal progenitor cells (NPCs), and facilitate neuronal transport, are significant to zebrafish neuroregeneration and neural repair (Kishimoto et al., 2012; Than-Trong and Bally-Cuif, 2015). In contrast to humans, zebrafish neuroregeneration is not inhibited by the formation of a glial scar due, in part, to the gene, *ctgfa*, which stimulates glial bridging (Mokalled et al., 2016). Categorized in the developmental process GO category, *ctgfa* was significantly up-regulated at 21 dpi (*p* < 0.0001; Fig. 6*A*). RGC marker *cxcl12a* was also significantly up-regulated at 21 dpi (*p* = 0.0095; Fig. 6*A*). In addition to regeneration, *cxcl12a* was categorized into regulation of cell motility and positive regulation of cellular differentiation GO clusters, wherein 100% and 91% of DEGs were up-regulated, respectively (Fig. 5). Within positive regulation of cellular differentiation, lysyl oxidase–like 2 genes, *loxl2a* and *loxl2b*, were also upregulated at 21 dpi (*p* < 0.0001; Fig. 6*A*,*B*). The *loxl2* genes are significant for neuronal repair, as loss of the *loxl2* genes has been shown to impair neural differentiation (Iturbide et al., 2015). Of the DEGs involved in neuroregeneration, half were selected for qPCR validation (Fig. 6*B*). Both RNA-seq and qPCR analysis revealed a similar increase in the upregulation of *apoeb*, *loxl2b*, and *notch1b* from 3 to 21 dpi.

## Discussion

With the high prevalence of mTBIs characterized by both short- and long-term cognitive effects, it is critical to develop an efficient, yet inexpensive, mTBI model that can be replicated in any laboratory. With DEGs categorized into GO clusters indicative of a peak injury response at 3 dpi and a peak neuroregeneration response at 21 dpi, this study validates a novel adult zebrafish mTBI model that requires minimal equipment in addition to a standard zebrafish aquatic housing system. In the efficient and effective setup, only a standardized ball bearing, clamped tube apparatus, and foam block cradle are required to adequately administer consistent mTBIs. This flexible model can also be adapted to observe the effects of differing traumatic brain injuries such as multiple, less severe impacts characteristic of chronic traumatic encephalopathy (Mouzon et al., 2014; Petraglia et al., 2014). Additionally, this novel model also has a behavioral assay that can measure spatial memory deficits in mTBI fish, an effect observed in other mTBI animal models and human patients (Lu et al., 2005; Lundin et al., 2006; Chen et al., 2012; Dawish et al., 2012; Luo et al., 2017).

Zebrafish make an excellent disease model because zebrafish and human brains share a high degree of homology, with 70% of human genes having at least one obvious zebrafish orthologue (Howe et al., 2013). The development of this mTBI model is especially significant, as it utilizes zebrafish, which share a strikingly similar genome to humans. This genomic similarity has important implications for the application of zebrafish neuroregeneration to the human brain. Additionally, the remarkable similarity, especially in the disease genome, between humans and zebrafish provides scientists with substantial research potential and promising pharmaceutical benefits (Lieschke and Currie, 2007). In accordance with the timelines observed in previous studies (Kishimoto et al., 2012; Kyritsis et al., 2012) as well as our previous data (not shown), GO categories in cell death and injury were expected at 3 dpi, while neuroregeneration and neural repair were expected at 21 dpi according to the novel zebrafish mTBI model. Specifically, response to cAMP was a significant GO cluster at 3 dpi (Table 3), which has been shown to promote neuronal survival (Hansen et al., 2003). Furthermore, up-regulation of the cAMP cascade has been shown to increase cellular proliferation (Nakagawa et al., 2002) and the number of new neurons (Zhu et al., 2004). MAP kinase tyrosine/serine/threonine phosphatase activity was also a GO category of interest at 3 dpi. Significantly, the MAP signaling pathway has been shown to induce neuritic outgrowth (Creedon et al., 1996) and is required for neuronal differentiation (Samuels et al., 2008). Within the MAP-kinase family are Jun N-terminal kinases (JNKs) that, in addition to differentiation, regulate cell proliferation and apoptosis (Dhanasekaran and Reddy, 2008) and have been identified as necessary for zebrafish tissue regeneration (Ishida et al., 2010). After the wound healing stage, JNKs help induce regeneration by phosphorylating Junb proteins (Ishida et al., 2010), the transcripts of which were found to be significantly upregulated at both 3 and 21 dpi (Fig. 4*A*). As evidenced by the enriched regulation of cell death GO category at 3 dpi (Fig. 3*A*), a primary response following mTBIs is apoptosis (Kroehne et al., 2011). To promote apoptosis, JNKs can also phosphorylate p53 proteins (Oleinik et al., 2007). Overexpression of p53 transactivates *dusp6*, which was significantly upregulated at 3 dpi (Fig. 4*A*), and induces cell death through inactivation of extracellular signal–regulated kinase 1/2 (ERK1/2; Piya et al., 2012). Apoptosis is also regulated by *dedd1*, a gene significantly upregulated at 3 dpi (Fig. 4*A*), which induces intermediate filament degradation (Lee et al., 2002).

Intermediate filaments are significant for maintaining cellular structure and facilitating transport and represent a significant GO cluster at 21 dpi (Table 4). Damaged intermediate filaments and other cellular structures are cleared by microglia or macrophages of the CNS. Markers for *apoeb*, for example, have been observed in microglia (Veth et al., 2011). Significantly, *apoeb* was upregulated at 21 dpi (Fig. 6*A*,*B*) and has been found to be involved in the wound healing process in both heart (Lien et al., 2006) and fin regeneration (Monnot et al., 1999; Poss et al., 2000). For regeneration, cellular differentiation is required to form new neurons that can ultimately be integrated into the site of injury. Within the positive regulation of cellular differentiation GO category enriched at 21 dpi, transcripts of the extracellular matrix proteins *loxl2a* and *loxl2b* were significantly upregulated (Fig. 6*A*,*B*). Expressed by NPCs (Maisel et al., 2007), the *loxl2* genes regulate pluripotency of embryonic stem cells (ESCs), and facilitate proper neural differentiation (Iturbide et al., 2015). The *loxl2* genes may also interact with the Notch 1 signaling pathway (Martin et al., 2015), which was significantly upregulated at 3 and 21 dpi as indicated by the *Notch1b* transcript expression. In proliferating cells of the ventricular zone (VZ), Notch 1 signaling has been shown to promote production of NPCs that can migrate toward the site of injury (Wang et al., 2009; Kishimoto et al., 2012). Migration is critical for regeneration, as indicated by the enriched regulation of cell motility GO category at 21 dpi (Fig. 5*C*). In response to cortical injury, Notch signaling has been observed in conjunction with an astrogliogenic response (Givogri et al., 2006). In mammals, astrogliosis results in the formation of an inhibitory glial scar not observed in zebrafish. Instead, *ctgfa*, which was significantly upregulated at 21 dpi (Fig. 6*A*), has been shown to induce glial bridging (Mokalled et al., 2016) where neuronal transport to the site of injury is ultimately facilitated by the filamentous RGCs. RGC marker *cxcl12a*, for example, was significantly upregulated at 21 dpi (Fig. 6*A*). With migration complete, new neurons can integrate and become fully functioning, mature neurons as indicated by the enriched neuroregeneration and neuron progenitor regeneration GO clusters at 21 dpi (Fig. 5).

Previous TBI studies in adult rodents have found changes in gene expression similar to those observed in this study. At 3 dpi, molecular activity within the MAP signaling pathway was significant (Table 3). Similarly, the MAP kinase cascade was found to be activated after injury in an adult rat weight-drop TBI model (Lu et al., 2015). Furthermore, postinjury quantification of newborn neurons in the hippocampus revealed increased neurogenesis after activation of the MAP signaling pathway (Lu et al., 2015). Within the MAP kinase family are JNKs that phosphorylate Junb proteins, which were upregulated at both 3 and 21 dpi (Fig. 4*A*). Increased Junb ipsilateral to the site of injury was also found after injury in a mild fluid percussion TBI model in rats (Raghupathi and McIntosh, 1996; Abrous et al., 1999).

At 21 dpi, genes specific to regeneration were differentially expressed, as observed in previous TBI studies. For example, the zebrafish transcript, *apoeb*, was significantly upregulated at 21 dpi (Fig. 6*A*,*B*). Likewise, in an adult rat study of parasagittal fluid percussion brain injury, an increase in ApoE mRNA expression was found around the cortical lesion site (Iwata et al., 2005). At both 3 and 21 dpi, *Notch1b*, involved in the Notch signaling pathway, was significantly upregulated (Fig. 6*A*,*B*). Similarly, a cortical stab wound injury model in mice found the Notch signaling pathway to be activated after injury (Givogri et al., 2006). More recently, postinjury upregulation of *Notch1* mRNA was also observed in a lateral fluid percussion injury model in rats (Puhakka et al., 2017). The glial bridge stimulating zebrafish transcript, *ctgfa*, which was significantly upregulated at 21 dpi (Fig. 6*A*), was also the focus of a weight-drop TBI model in rats. In the rat TBI study, a significant increase in non-neuron CTGF^+^ cells was observed at and around the lesion site over time (Liu et al., 2014).

The results of this study are two-fold: (1) the establishment and validation of a novel adult zebrafish mTBI model, and (2) the identification of significant genes and pathways involved in zebrafish CNS injury and neuroregeneration. The introduction of this effective, yet inexpensive, zebrafish mTBI model will significantly benefit the neuroscience community by providing greater access to study of zebrafish response to injury. In the future, additional sequencing depth may provide sufficient statistical power to identify additional differentially regulated genes involved in the response to mTBI. This same model may be used to look at additional time points, either to analyze the immediate changes in gene expression closely following injury or to longitudinally follow the neurorecovery process further. Ultimately, understanding the genetic basis of zebrafish neuroregeneration will help elucidate therapeutic targets for neural repair in humans.

## Synthesis

Reviewing Editor: Jun Chen, University of Pittsburgh Decisions are customarily a result of the Reviewing Editor and the peer reviewers coming together and discussing their recommendations until a consensus is reached. When revisions are invited, a fact-based synthesis statement explaining their decision and outlining what is needed to prepare a revision will be listed below. The following reviewer(s) agreed to reveal their identity: Wen Zhu, Samuel Newton

Both reviewers appear to be happy about this revision, and most of their previous concerns have been addressed with rigor. Their remaining concerns are minor, wich are provided below.

Reviewer #1

Current presentations of all tables are confusing and difficult to read. It would be appropriate if authors could revise them with a better format to increase the readability.

Reviewer #2

The authors have improved the confidence of the gene expression data by performing QPCR based secondary validation. Additionally, testing the effect of mTBI using a spatial memory behavioral paradigm and obtaining meaningful results suggests that this zebra fish model can be developed for further study in the field.

A minor concern: The Abstract captures essential features of the study but appears to be missing the behavioral component.
